# Association between participation restriction due to hearing loss and self-perception of health, social support, and quality of life in elderly people: a cross-sectional study

**DOI:** 10.1590/2317-1782/20242023299en

**Published:** 2024-09-02

**Authors:** Anna Clara Simon Landim Silveira, Marisa Silvana Zazzetta, Fabiana de Souza Orlandi, Sofia Cristina Iost Pavarini, Ariene Angeline dos Santos, Karina Gramani Say, Isabela Thaís Machado de Jesus, Grace Angélica de Oliveira Gomes, Aline Cristina Martins Gratão, Letícia Pimenta Costa-Guarisco

**Affiliations:** 1 Departamento de Gerontologia, Universidade Federal de São Carlos – UFSCar - São Carlos (SP), Brasil.; 2 Departamento de Enfermagem, Universidade Federal de São Carlos – UFSCar - São Carlos (SP), Brasil.

**Keywords:** Aged, Aging, Hearing Loss, Social Support, Quality of Life, Health of the Elderly

## Abstract

**Purpose:**

To verify the association between participation restriction due to hearing loss and self-perception of health, social support, and quality of life in elderly people.

**Methods:**

This is a cross-sectional, observational, and descriptive study with a quantitative data approach. A database with information collected in a medium-sized Brazilian municipality was used. The study was conducted with 235 elderly people registered in five Family Health Strategy Units. Sociodemographic and health information and the results of the following questionnaires were used: Hearing Handicap Inventory for the Elderly – Screening Version (HHIE-S), Medical Outcomes Study (MOS) Social Support Survey, Subjective Health Assessment, and Short-Form 6-Dimension (SF-6D) Health and Quality of Life Index. Groups with and without participation restriction were compared according to sociodemographic, health, social support, and quality of life variables. A multivariate binary logistic regression method was employed to evaluate the associations between the independent variables and participation restriction.

**Results:**

The group with participation restriction is composed of older individuals with lower quality of life and poorer self-perception of health. Poorer self-perception of health was the only predictor of participation restriction related to hearing loss.

**Conclusion:**

Participation restriction is associated with poorer self-perception of health. The study highlights the importance of assessing individuals’ self-perception regarding biopsychosocial issues, in addition to considering the environmental context to understand the social and emotional impacts caused by hearing loss.

## INTRODUCTION

The concept of healthy aging, as advocated by the World Health Organization (WHO), is defined as “the process of developing and maintaining the functional ability that enables well-being in older age. (p. 28)” Functional ability corresponds to the interaction between an individual’s intrinsic capacities and their environment. Intrinsic capacities, in turn, refer to a set of physical and mental abilities related to health, including sensory abilities as one of its components^(^1^)^. This means that, in the light of healthy aging, auditory conditions are determinants for intrinsic capacities, which, together with the individual’s interactions with their environment, will determine their functional ability.

Sensory losses, whether auditory or visual, are chronic non-communicable conditions resulting from the human aging process. However, their existence does not necessarily express the actual impact they may have on individuals’ lives^(^1^)^. A complexity of domains interferes with a person’s functional ability. For this reason, in 2001, the WHO proposed the International Classification of Functioning, Disability, and Health (ICF), which is a model for classifying categories related to health conditions that assists in understanding changes in body function and structure and levels of capacity and performance, the latter being determined by the usual environment in which the individual lives. These categories allow for the gathering of information from the perspectives of the body (Body Functions and Structures), the individual, and social aspects (Activities and Participation). Thus, the ICF enabled a redesign of health care by describing the functionality of people with certain health conditions, as well as disability, measured by limitations in daily activities and restrictions on participation in the social and environmental context^(^2^)^.

The consequences of hearing impairment vary from individual to individual and are influenced by factors such as health, ability to adapt to different situations, life experiences, among others. Therefore, people with similar auditory characteristics can have different levels of participation restriction, and there is not necessarily a direct association between the degree of loss and disability, as this depends on the interactions between the individual and their environment^(^3,4^)^. The emotional and social effects of hearing loss are conditions that vary according to the individual’s perception of their participation restriction, and can be influenced by health-related issues, quality of life, and social demands^(^5-8^)^. Thus, subjective assessment plays a crucial role in understanding the impacts related to auditory and socioemotional conditions, as well as health perception, which is a useful indicator of individual well-being conditions^(^9^)^.

Health self-perception—although subjective—is a relevant indicator for understanding an individual’s view of their health and well-being, as it encompasses both biological and psychosocial issues^(^10,11^)^. In the literature, poor health self-perception in older people with hearing loss is prevalent and is related to other elements such as depression, dependency for daily activities, and social participation restriction resulting from the limitation caused by hearing loss^(^7,11^)^.

Social support networks are of great importance to elderly people with hearing loss for establishing communication networks and reducing loneliness, and are considered the greatest predictor of social issues and quality of life^(^12-15^)^. The environment and its interactions, often represented by family, friends, or neighbors, are determinants for the intrinsic capacity of older people and act as favorable factors or barriers to functional ability^(^1^)^. It is worth noting that, because of hearing difficulties, family and friends may restrict communication with older people to only essential matters because they lack the skills to handle the difficulty, leading to disadvantages such as reduced social engagement and loneliness of these people, which is a risk factor for mortality and worsening health conditions^(^16,17^)^. These aspects impact the quality of life of individuals^(^5,12^)^.

Quality of life, in turn, encompasses various factors such as individual well-being, functional capacity, social interaction, satisfaction, emotional state, and subjective influences^(^18-20^)^. It can be defined as an individual’s perception of their position in life, influenced by their goals, expectations, standards, and concerns, considering their cultural context and values^(^21^)^. Studies highlight the relationship between the consequences and negative impact of hearing loss on the quality of life of the elderly population^(^6,12,15^)^. It is evident that the environmental domain of quality of life, which is linked to opportunities, leisure, physical environment, and safety, is highly relevant to participation restriction related to hearing loss^(^8^)^.

There are numerous studies on factors associated with hearing loss, mostly using information on auditory conditions obtained through tonal audiometry, which only investigates the existence of hearing impairment^(^13,22^)^. However, auditory participation restriction is a condition of individual self-perception and does not have a direct relationship with the degree of hearing loss. In other words, individuals with hearing loss have different perceptions of participation restriction, which are not always justified by the hearing impairment measured by audiometry^(^5^)^.

Thus, given the issues raised in the study and according to the ICF, there is a need to understand more comprehensively how biopsychosocial and contextual factors can impact participation restriction and, consequently, healthy aging, considering the functional capacity of the elderly population. These issues may be useful in clarifying the reasons for the failures related to hearing rehabilitation in this population, as audiological characteristics alone are not sufficient to understand the difficulties presented in the adaptation of hearing aids.

Therefore, this study aimed to verify whether the subjective evaluation of health, social support, and quality of life are factors associated with participation restriction due to hearing loss in elderly people.

## METHODS

### Study design, location, and sample

This is a cross-sectional, observational, and descriptive study with a quantitative data approach. The project was reviewed by the Research Ethics Committee of the affiliated university (CAAE: 86967418.4.0000.5504) and approved under opinion no. 3.101.282, respecting the ethical aspects provided by Resolution 510/2016 regulated by the National Health Council.

Data collected between 2017 and 2018 from a community in a highly socially vulnerable context of a medium-sized city (approximately 250,000 inhabitants) were used, originating from the study “Monitoring tool for levels of frailty in elderly people attended in Primary Health Care: assessment of its effectiveness and efficiency” (Opinion no. 2.424.616/2017, CAAE: 66076017.3.0000.5504) by the Management and Aging Research Group, funded by the São Paulo Research Foundation – FAPESP, conducted between 2017 and 2018.

Before the start of data collection, a sample calculation was performed to ensure sample representativeness. First, contact was made with the teams of the Family Health Units (USF) to present the research, followed by contact with the elderly people registered in the Units. Initial contact was made through home visits, where they were informed about the study and invited to participate by signing an Informed Consent Form (ICF). After the participants' written consent, data collection was scheduled at home and conducted by previously trained researchers.

A total of 238 elderly people registered in five USFs, assisted free of charge by the Brazilian government, were evaluated in their homes. Inclusion criteria were age ≥60 years, registration with a USF assisted by the Family Health Support Center (NASF), and the ability to understand and communicate verbally. Exclusion criteria included conditions that prevented testing, such as severe motor or cognitive deficits previously diagnosed or reported by family members, wheelchair use, or terminal illness.

The study sample comprised 235 elderly people, with three excluded from the database because of a lack of information regarding the variable auditory participation restriction.

### Measures and variables

Sociodemographic and health data were obtained from participants through the administration of a self-reported questionnaire prepared by the researchers. The data included information on sex (male and female), age in years, ethnicity (self-declared skin color), marital status (with and without a partner), education (in years), and the presence of self-reported comorbidities (yes or no) such as systemic arterial hypertension, diabetes mellitus, cancer, osteoporosis, and stroke.

### Auditory assessment: participation restriction

The Hearing Handicap Inventory for the Elderly – Screening Version (HHIE-S) questionnaire was used in this study to assess participation restriction related to hearing loss in older people, in its social and emotional aspects^(^23^)^. The questionnaire was translated and validated for use in Brazilian Portuguese^(^24^)^. Composed of ten questions, it offers three response options: yes (4 points), sometimes (2 points), and no (0 points). The total score is calculated by summing the scores for each question. Values above 8 indicate participation restriction, and the higher the score, the greater the restriction.

### Subjective health assessment

The Subjective Health Assessment consisted of five questions aimed at verifying the elderly individual’s self-perception of their own health^(^25^)^. Each question was assigned a score ranging from 1 to 3 points. The total score was calculated by summing the scores for each question, with a higher score indicating a better self-assessment of health.

### Social support assessment

The social support instrument from the Medical Outcomes Study (MOS) was used in its translated and validated Portuguese version^(^26^)^. The MOS Social Support Survey consists of 20 questions that refer to the support or help received by the individual, according to their own perception, including material, instrumental, affective, informational, and social interaction support. The respondent should consider the frequency at which they receive each type of support, with options: never (1 point), rarely (2 points), sometimes (3 points), almost always (4 points), and always (5 points). Scores for each type of support range from 20 to 100 points, with higher scores indicating a higher level of social support^(^27^)^.

### Quality of life assessment

The Short-Form 6-Dimension (SF-6D) Health and Quality of Life Index instrument was used to assess quality of life, a generic questionnaire developed in the United Kingdom from the simplification and reduction of the Short-Form 36 (SF-36)^(^28^)^. This study uses the most updated version of the SF-6D, validated in Brazil^(^29^)^. Its purpose is to evaluate health status through the six dimensions it comprises: functional capacity, global limitation, social aspects, pain, mental health, and vitality. Each of these dimensions has a set of four to six alternatives. Scores range from zero to one and correspond to the person’s preference strength for a particular health state—where “zero” refers to the worst and “one” to the best health state^(^29-32^)^.

### Statistical analyses

Continuous variables were presented as mean and standard deviation, and the groups were compared using the Student’s *t*-test or Mann-Whitney test, depending on the result of the Shapiro-Wilk test. For categorical variables, absolute and relative frequencies were obtained, and the groups were compared using the Pearson’s Chi-squared test.

A Binary Logistic Regression Model was used to evaluate participation restriction, involving subjective health, quality of life, and social support assessments, and considered sociodemographic characteristics such as age, sex, educational level, and the presence of comorbidities as covariates. The most appropriate model was chosen using the stepwise progression strategy as the variable selection criterion. The modeling results are presented as odds ratios with corresponding 95% confidence intervals.

All analyses were conducted using the Statistical Package for the Social Sciences (SPSS), version 21.0.

## RESULTS

Participation restriction was analyzed according to sociodemographic, health, quality of life, and social support variables (Table 1). Variables that showed a statistically significant relationship with auditory participation restriction included age, quality of life, and self-perception of health. The group with participation restriction is composed of older individuals with lower quality of life and poorer self-perception of health compared to the group without participation restriction.

**Table 1:** Sociodemographic and health variables, subjective health assessment, social support, and quality of life according to participation restriction due to hearing loss

**Variable**	**N**	**Without Participation Restriction**	**N**	**With Participation Restriction**	***p*-value**
Female sex ^1^	100	58.10%	36	57.10%	0.891
Male sex ^1^	72	41.90%	27	42.90%	
Age (in years) 2	172	71.04 (±6.60)	63	74.29 (±8.58)	0.008
Educational level (in years) 3	172	2.55 (±2.73)	63	3.03 (±3.06)	0.251
Marital status – married or with partner ^1^	102	61.10%	36	59.00%	0.778
Household income (in reais)^3^	135	2252.00 (±1363.08)	41	1913.56 (±889.71)	0.137
Presence of comorbidities 1	78	45.30%	26	41.30%	0.577
Quality of life ^3^	171	0.76 (±0.13)	61	0.72 (±0.15)	0.041
Social support ^3^	170	81.98 (±17.88)	59	80.66 (±19.59)	0.634
Health self-perception ^3^	169	7.87 (±2.28)	60	6.76 (±2.34)	0.002

1 Pearson’s Chi-Squared Test;

2 Mann-Whitney Test;

3 Student’s *t*-Test

Analysis of the variables associated with participation restriction showed that the model containing only the subjective health assessment is the only significant one [*x* ^2^ (1) = 4.20; p < 0.04, Nagelkerke *R* ^2^ = 0.04], indicating that poorer self-perception of health is a predictor of participation restriction related to hearing loss (OR = 0.85; 95% CI = 0.72 - 0.99).

## DISCUSSION

It is understood that hearing loss worsens with aging, and older individuals have a higher prevalence of participation restriction due to communication difficulties and reduced social participation^(^33,34^)^. Two out of three individuals with any level of hearing loss will develop significant participation restriction within five years^(^33^)^. Thus, it is important to understand the factors related to this condition, as hearing loss is prevalent and progressive in the elderly population, with potentially negative outcomes for aging.

Studies have shown that poorer quality of life is related to hearing difficulties and participation restriction^(^8,12,33-35^)^. Quality of life assessment is associated with multiple personal (internal) and environmental (external) aspects: the stability of interpersonal relationships, good health status, and the person's ability to adapt to their environment^(^33^)^. Therefore, when hearing loss leads to participation restriction, it compromises well-being, can cause stress and loneliness, and impacts quality of life^(^5,36,37^)^. Elderly people with participation restriction are more likely to present lower quality of life, especially related to environmental factors^(^8^)^. According to the ICF, contextual factors, including environmental and personal aspects, can act as facilitators or barriers to a person's functionality and disability, highlighting the importance of considering contextual factors when assessing participation restriction and understanding the individual as a whole in their environment^(^2^)^.

A previous study^(^34^)^ analyzed the association between participation restriction due to hearing loss and quality of life in a sample of older people (781 men and 950 women). The quality of life test considered subjective well-being, depressive symptoms, loneliness, and physical functionality. After controlling for risk factors for presbycusis, these authors demonstrated that quality of life was significantly associated with participation restriction, as evaluated by the HHIE-S, but not with a single question about the existence of hearing problems. Additionally, among the set of variables that comprised the quality of life assessment, subjective well-being was most strongly associated with participation restriction in elderly people, with an odds ratio of 4.6 (95% CI: 2.9 - 7.5), showing that poor health perception considerably increases the chances of participation restriction^(^34^)^, corroborating the results of the present study.

The association between subjective well-being and hearing impairment was also identified in another earlier study^(^34^)^. Controlled for different variables such as age, gender, health conditions, and hospitalization in the past year, participation restriction due to hearing loss was associated with a higher likelihood of having a poorer self-perception of health, indicating an independent association between participation restriction and well-being indicators. This result, like that of the present research, demonstrates that general health, as perceived by the individual, influences their communication, interaction, and social participation skills.

Subjective health assessment has proven to be an important indicator of well-being in elderly populations, with the potential to influence other aspects of their lives^(^10,38,39^)^. According to the WHO, this complexity in the health and functionality states of older people raises fundamental questions about what is meant by health in older age, how it is measured, and how it can be promoted. Comprehensive assessments of these health states are better predictors of negative outcomes than the presence of individual diseases or even the degree of comorbidities. Therefore, they serve as a population health indicator, considered one of the vital measures for evaluating health outcomes^(^40^)^.As aging progresses, individuals become more susceptible to physical, psychosocial, physiological, and cognitive losses, which reflect on their health outcomes and contribute to a negative perception of these changes compared to their younger years^(^41^)^. The emotional and social factors encompassing the evaluation of participation restriction due to hearing loss are highly relevant to the physical and mental health of elderly people and impact their self-assessment of health^(^33,42^)^, sense of autonomy, control, and functionality in daily activities, as well as their overall health perception. Understanding the complex interactions between health self-assessment and the dimensions that influence changes in health perception can be key to the well-being of elderly individuals^(^43,44^)^.

It is important to take a holistic view of the individual, considering that they are in a different phase and context of life. Their perception of their own health and subjective well-being are relevant mechanisms for health professionals to assist with the choice of interventions to address the changes that occur with aging.

The literature indicates that socioeconomic factors interfere with the perception of participation restriction^(^8^)^. Contrarily, in this study, age, educational level, income, and social support did not differ between groups with and without participation restriction. It is noteworthy that the sample comprised socially vulnerable elderly individuals with very similar socioeconomic characteristics, which may explain the similarity between the studied groups. However, conducting this study with an elderly population in a context of high social vulnerability adds a differential to the literature, as participation restriction is also influenced by the environment in which the person lives. The impacts resulting from hearing impairment are also products of the conditions imposed by the environment, access opportunities, and health conditions^(^45^)^.

This study highlights the importance of self-perception in the biopsychosocial evaluation of older people. Another relevant factor is that the research is based on participation restriction due to hearing loss, considering that audiometric tests do not assess the environmental context or how individuals feel about the emotional and social aspects of communication. Finally, it points out that the HHIE-S is a more reliable, specific, and sensitive instrument for evaluating the impact of hearing loss on quality of life compared to the single question, “Do you have hearing loss?”^(^35^)^. Thus, the importance of using this instrument to detect the impacts of hearing loss is emphasized.

Assessing an individual’s intrinsic capacities and their relationship with the environment is fundamental to understanding functional capacity and its impacts on the healthy aging process. Therefore, self-perception of health and communication aspects can be useful for actions aimed at biopsychosocial health and should be considered by a multidisciplinary team. This study highlights the importance of further research to understand the relationship between subjective well-being and perceptions of hearing loss as valuable information in the auditory rehabilitation processes of the elderly population, favoring the use, benefit, and satisfaction with hearing amplification devices.

A limitation of the present study lies in the sample being drawn from a single Brazilian city, involving a community with very similar social characteristics. On the other hand, there is a differential in this respect, as it encompasses a home-based sample in a situation of social vulnerability, within a universe of Brazilian research predominantly conducted in outpatient settings or field research carried out in wealthy countries with different population characteristics. Thus, understanding the results from elderly people living in socially vulnerable communities is more faithful to reality and allows for actions that foster equity.

## CONCLUSION

In this study, the only variable independently associated with participation restriction due to hearing loss in elderly individuals was subjective health assessment. Although quality of life was lower in the group with participation restriction, this association did not remain significant when controlling for other variables. Regarding social support, no relationship with participation restriction was found. The study’s relevance integrates the individual’s self-perception of their health conditions and the impact of hearing loss, with a focus on participation restriction.
