# Supplementary material for: Association between participation restriction due to hearing loss and self-perception of health, social support, and quality of life in elderly people: a cross-sectional study
Source: Codas. 2024 Sep 2;36(5):e20230299. doi: 10.1590/2317-1782/20242023299en (PMC11404837; doi:10.1590/2317-1782/20242023299en)
Supplement: Translation

# Translation: Associação entre restrição à participação decorrente de perda auditiva e autopercepção de saúde, suporte social e qualidade de vida da pessoa idosa: estudo transversal

**DOI:** 10.1590/2317-1782/20242023299pt

**Authors:** Anna Clara Simon Landim Silveira, Marisa Silvana Zazzetta, Fabiana de Souza Orlandi, Sofia Cristina Iost Pavarini, Ariene Angeline dos Santos, Karina Gramani Say, Isabela Thaís Machado de Jesus, Grace Angélica de Oliveira Gomes, Aline Cristina Martins Gratão, Letícia Pimenta Costa-Guarisco

**Affiliations:** 1 Departamento de Gerontologia, Universidade Federal de São Carlos – UFSCar - São Carlos (SP), Brasil.; 2 Departamento de Enfermagem, Universidade Federal de São Carlos – UFSCar - São Carlos (SP), Brasil.

**Keywords:** Idoso, Envelhecimento, Perda Auditiva, Apoio Social, Qualidade de Vida, Saúde do Idoso

## Abstract

**Objetivo:**

Verificar a associação entre a restrição à participação decorrente de perda auditiva e a autopercepção da saúde, do suporte social e da qualidade de vida em pessoas idosas.

**Método:**

Estudo com delineamento transversal, observacional, descritivo e com abordagem quantitativa dos dados. Foi utilizado um banco de dados com informações coletadas em um município brasileiro de médio porte, sendo incluídas no estudo 235 pessoas cadastradas em cinco unidades de Estratégia de Saúde da Família. Utilizou-se informações sociodemográficas, de saúde e os resultados dos questionários: avaliação à restrição à participação (Hearing Handicap Inventory for the Elderly Screening Version - HHIE-S), Suporte Social (Escala de Apoio Social do MOS), Avaliação Subjetiva de Saúde e a Qualidade de Vida (Short-Form 6 dimensions- SF-6D). Comparou-se os grupos com restrição e sem restrição à participação segundo as variáveis sociodemográficas, de saúde, suporte social e qualidade de vida. Um método de regressão logística binária multivariado foi utilizado para avaliar as associações entre as variáveis independentes e a restrição à participação.

**Resultados:**

O grupo com restrição à participação é mais velho, possui menor qualidade de vida e pior autopercepção de saúde. Esta se mostrou ser o único preditor da restrição à participação relacionada à perda auditiva.

**Conclusão:**

A restrição à participação está associada a uma pior autopercepção de saúde. O estudo revela a importância de avaliar a autopercepção dos indivíduos quanto às questões biopsicossociais, além de considerar o contexto ambiental para a compreensão dos impactos sociais e emocionais da perda auditiva.

## INTRODUÇÃO

O conceito de envelhecimento saudável preconizado pela Organização Mundial de Saúde (OMS) é definido como “o processo de desenvolvimento e manutenção da capacidade funcional que permite o bem-estar em idade avançada (p. 28)”. A capacidade funcional corresponde à interação entre as capacidades intrínsecas do indivíduo com o seu ambiente. Por sua vez, capacidades intrínsecas referem-se ao conjunto das capacidades físicas e mentais, possuindo relação com a saúde, incluindo aqui as habilidades sensoriais como um de seus componentes^(1)^. Isso quer dizer que, à luz do envelhecimento saudável, as condições auditivas são determinantes para as capacidades intrínsecas que, juntamente com as interações do sujeito com o seu ambiente, determinarão a sua capacidade funcional.

As perdas sensoriais, auditivas ou visuais, são condições crônicas não transmissíveis decorrentes do processo de envelhecimento humano, contudo, a sua existência não expressa de fato o que elas podem causar de impacto na vida dos indivíduos^(1)^. Uma complexidade de domínios interferem na capacidade funcional de uma pessoa. Por esse motivo a OMS, em 2001, propõe a Classificação Internacional de Funcionalidade, Incapacidade e Saúde (CIF) que é um modelo de classificação de categorias relacionadas às condições de saúde que nos ajudam a entender mudanças na função e estrutura do corpo, seu nível de capacidade e de desempenho, sendo este último determinado pelo ambiente habitual em que o indivíduo vive. Essas categorias permitem a obtenção de informações nas perceptivas corporal (Função e Estrutura do Corpo), individual e social (Atividades e Participação). Assim, a CIF permitiu um redimensionamento do cuidado em saúde ao descrever a funcionalidade de pessoas com determinadas condições de saúde, bem como a incapacidade, dimensionada pela limitação nas atividades do cotidiano e a restrição à participação no contexto social e ambiental^(2)^.

As consequências geradas pela deficiência auditiva variam de indivíduo para indivíduo e têm influência de fatores como a saúde, capacidade de adaptação em situações diversas, vivências ao longo da vida, dentre outras. Sendo assim, pessoas com semelhantes características auditivas podem ter diferentes níveis de restrição à participação e não há, necessariamente, uma associação direta entre o grau da perda e a incapacidade, pois esta depende das interações entre o indivíduo e seu ambiente^(3,4)^. Os efeitos emocional e social da perda auditiva são condições que variam de acordo com a percepção individual do sujeito sobre sua restrição à participação e podem ser influenciadas por questões relacionadas à sua saúde, sua qualidade de vida e demandas sociais^(5-8)^. Desse modo, a avaliação subjetiva possui um papel fundamental para a compreensão dos impactos voltados às condições auditivas e socioemocionais, bem como à percepção da saúde que é um indicador útil das condições de bem-estar individual^(9)^.

A autopercepção de saúde, apesar de subjetiva, é um indicador relevante para depreender a visão do indivíduo sobre o seu estado de saúde e bem-estar, pois compreende tanto questões biológicas quanto psicossociais^(10,11)^. Na literatura, a autopercepção de saúde ruim em pessoas idosas com perda auditiva mostra-se prevalente e está relacionada a outros elementos como depressão, dependência para atividades diárias e restrição à participação social decorrente da limitação causada pela perda auditiva^(7,11)^.

A rede de apoio social é de grande importância às pessoas idosas com perda auditiva para o estabelecimento da rede de comunicação e diminuição da solidão, além de ser considerada o maior preditor da questão social e de qualidade de vida^(12-15)^. O ambiente e suas interações, muitas vezes representado pela família, amigos ou vizinhos, são determinantes para a capacidade intrínseca da pessoa idosa e atuam como fatores favoráveis ou barreiras da capacidade funcional^(1)^. Salienta-se que devido às dificuldades auditivas, familiares e amigos podem restringir a comunicação com a pessoa idosa apenas às questões essenciais por não apresentarem habilidade para lidar com sua dificuldade, acarretando em prejuízos como a diminuição do engajamento social da pessoa idosa e a solidão, a qual é um risco para a mortalidade e à piora do estado da saúde^(16,17)^. Destaca-se que esses aspectos apresentados impactam na qualidade de vida dos indivíduos^(5,12)^.

A qualidade de vida, por sua vez, abarca diversos fatores tais como o bem-estar individual, capacidade funcional, interação social, satisfação, estado emocional e possui influências subjetivas^(18-20)^. Ela pode ser definida como a percepção do indivíduo sobre sua posição na vida e é influenciada por seus objetivos, expectativas, padrões e preocupações, considerando seu contexto cultural e valores^(21)^. Estudos expressam a relação das consequências e o impacto negativo da perda auditiva na qualidade de vida da população idosa^(6,12,15)^. Evidencia-se que o domínio ambiental da qualidade de vida, o qual está ligado às oportunidades, lazer, ambiente físico e segurança, apresenta uma alta relevância na restrição à participação relacionada à perda auditiva^(8)^.

Na literatura encontram-se inúmeros estudos sobre fatores associados às perdas auditivas utilizando, em sua maioria, informações das condições auditivas obtidas por meio da audiometria tonal, a qual investiga apenas a existência da incapacidade auditiva^(13,22)^. Já a restrição à participação auditiva é uma condição de autopercepção individual e que não mantém relação direta com o grau da perda auditiva, ou seja, indivíduos com perdas auditivas possuem diferentes percepções da restrição à participação, que nem sempre se justificam pela incapacidade auditiva aferida pela audiometria^(5)^.

Assim, perante às questões trazidas no estudo e de acordo com a CIF, evidencia-se a necessidade de compreender melhor como os fatores biopsicossociais e contextuais podem impactar a restrição à participação e portanto, o envelhecimento saudável considerando a capacidade funcional da pessoa idosa. Essas questões podem ser úteis para esclarecer os motivos dos insucessos relacionados à reabilitação auditiva na população idosa, uma vez que as características audiológicas não são suficientes para compreender as dificuldades apresentadas na adaptação de próteses auditivas.

Diante disso, o estudo visou verificar se a avaliação subjetiva de saúde, do suporte social e da qualidade de vida são fatores associados à restrição à participação relacionada à perdas auditivas em pessoas idosas.

## MÉTODO

### Desenho, local do estudo e amostra

Trata-se de um estudo com delineamento transversal, observacional, descritivo e com abordagem quantitativa dos dados. O projeto foi apreciado pelo Comitê de Ética em Pesquisa da universidade vinculada (CAAE: 86967418.4.0000.5504) e aprovado conforme parecer nº 3.101.282 respeitando os aspectos éticos previstos pela Resolução 510/2016 regulamentada pelo Conselho Nacional de Saúde.

Foram utilizados dados coletados no período entre 2017 e 2018, em uma comunidade em contexto de alta vulnerabilidade social de uma cidade médio porte (aproximadamente 250 mil habitantes), provenientes do estudo “Ferramenta de Monitoramento de Níveis de Fragilidade em Idosos Atendidos na Atenção Básica de Saúde: avaliação de sua efetividade e eficiência” (Parecer nº. 2.424.616/2017, CAAE: 66076017.3.0000.5504) do Grupo de Pesquisa “Gestão e Envelhecimento” sob fomento da Fundação de Amparo à Pesquisa do Estado de São Paulo - FAPESP entre 2017 a 2018.

Antes do início da coleta, foi feito o cálculo amostral para garantir a representatividade da amostra. Primeiramente foi realizado o contato com as equipes das Unidades de Saúde da Família (USFs) para apresentação da pesquisa e posteriormente com as pessoas idosas cadastradas nas Unidades. O contato inicial foi efetuado por intermédio de visitas domiciliares, nas quais foram informados sobre a pesquisa e convidados a participarem por meio da assinatura do Termo de Consentimento Livre e Esclarecido (TCLE). Após o consentimento escrito dos participantes, foi agendada a coleta de dados em domicílio, realizada por pesquisadores previamente treinados.

Foram avaliadas, em seus domicílios, 238 pessoas idosas cadastradas em cinco unidades da Estratégia de Saúde da Família, assistidas gratuitamente pelo governo brasileiro. O estudo teve como critérios de inclusão idade igual ou superior a 60 anos, ser cadastrado em uma USFs assistida pelo Núcleo de Apoio à Saúde da Família (NASF), ter capacidade de compreensão e comunicação verbal. Os critérios de exclusão foram condições que impediam a realização dos testes como déficits motores ou cognitivos graves previamente diagnosticados ou relatados por familiares, uso de cadeira de rodas ou doenças em estado terminal.

A amostra deste estudo foi composta por 235 pessoas idosas, sendo 3 excluídas do banco de dados devido à falta de informação a respeito da variável auditiva restrição à participação.

### Medidas e variáveis

Dados sociodemográficos e de saúde: foram obtidos dos participantes por meio de um questionário autorreferido construído pelos pesquisadores. Os dados incluíram informações sobre sexo (masculino e feminino), idade em anos, etnia (cor da pele autodeclarada), estado civil (com e sem parceiro), escolaridade (em anos) e presença de comorbidades autodeclaradas (sim ou não) - como hipertensão arterial sistêmica, *diabetes mellitus*, câncer, osteoporose e acidente vascular cerebral.

### Avaliação auditiva: restrição à participação

O questionário *Hearing Handicap Inventory for the Elderly - Screening* (HHIE-S), foi utilizado neste estudo para avaliar a restrição à participação relacionada à perda auditiva em pessoas idosas, em seus aspectos social e emocional^(23)^. O questionário foi traduzido e validado para uso em português brasileiro^(24)^. Composto por dez questões, oferece três opções de resposta: sim (4 pontos), às vezes (2 pontos) e não (0 pontos). A pontuação total é calculada somando as pontuações em cada pergunta. Valores superiores a 8 são indicativos de restrição à participação e, quanto maior a pontuação, maior a restrição.

### Avaliação subjetiva de saúde

A Avaliação Subjetiva de Saúde foi composta por 5 perguntas que visam verificar a autopercepção do indivíduo idoso sobre sua própria saúde^(25)^. Para cada pergunta foi atribuída uma pontuação, que variou de 1 a 3 pontos. A pontuação total foi calculada somando as pontuações em cada pergunta, sendo que quanto maior a pontuação, melhor a autoavaliação da saúde.

### Avaliação do suporte social

Foi utilizado o instrumento de apoio social do *Medical Outcomes Study* (MOS) na versão traduzida e validada para a Língua Portuguesa^(26)^. A Escala de Apoio Social do MOS é constituída por 20 perguntas que referem-se ao apoio ou a ajuda recebida pelo indivíduo, segundo sua própria percepção, incluindo o apoio material, instrumental, afetivo, de informação e de interação social. O entrevistado deve considerar a frequência com que conta com cada um dos apoios, sendo composto pelas opções: nunca (1 ponto), raramente (2 pontos), às vezes (3 pontos), quase sempre (4 pontos) e sempre (5 pontos). Os escores para cada tipo de apoio variam de 20 a 100 pontos e, quanto maior a pontuação, maior o nível de apoio social^(27)^.

### Avaliação da qualidade de vida

Para a avaliação da qualidade de vida foi utilizado o instrumento S*hort-Form 6 dimensions* (SF-6D), que é um questionário genérico desenvolvido no Reino Unido, a partir da simplificação e redução do *Short-Form 36* (SF-36)^(28)^. O presente estudo utiliza a versão mais atualizada do SF-6D, validada no Brasil^(29)^. Sua finalidade é avaliar o estado de saúde por meio das seis dimensões que o compõem: capacidade funcional, limitação global, aspectos sociais, dor, saúde mental e vitalidade. Em cada uma dessas dimensões há um conjunto de quatro a seis alternativas. A pontuação varia de zero a um e corresponde à força da preferência da pessoa por um determinado estado de saúde - sendo que “zero” refere-se ao pior e “um” o melhor estado de saúde^(29-32)^.

### Análises estatísticas

As variáveis contínuas foram apresentadas por meio de média e desvio padrão e os grupos comparados por meio do Teste T de Student ou de Mann-Whitney, dependendo do resultado do Teste de Shapiro-Wilk. Para as variáveis categóricas obteve-se a frequência absoluta e relativa e os grupos comparados por meio do Teste Qui-quadrado de Pearson.

Um Modelo de Regressão Logística Binária foi utilizado para avaliar a restrição à participação, envolvendo a avaliação subjetiva de saúde, qualidade de vida e suporte social, e considerou como covariáveis características sociodemográficas, como idade, sexo, anos de escolaridade, além da presença de comorbidades. O modelo mais adequado foi escolhido usando a estratégia *stepwise* de progressão como critério de seleção de variável. Os resultados da modelagem são apresentados como *Odds Ratios* com correspondentes intervalos de confiança de 95%.

Para todas as análises foi utilizado o programa *Statistical Package for the Social Sciences* (SPSS), versão 21.0.

## RESULTADOS

A restrição à participação foi analisada segundo as variáveis sociodemográficas, de saúde, qualidade de vida e suporte social (Tabela 1)*.* Destarte, há variáveis que revelaram relação estatisticamente significante com a restrição à participação auditiva, sendo estas: idade, qualidade de vida e a autopercepção de saúde. O grupo com restrição à participação é mais velho, com pior qualidade de vida e pior autopercepção da saúde comparado ao grupo sem restrição à participação.

**Tabela 1:** Variáveis sociodemográfica e de saúde, avaliação subjetiva de saúde, suporte social e qualidade de vida segundo a restrição à participação decorrente de perda auditiva auditiva

**Variáveis**	**N**	**Sem restrição à Participação**	**N**	**Com restrição à Participação**	**P – Valor**
Sexo Feminino^1^	100	58,10%	36	57,10%	0,891
Sexo Masculino^1^	72	41,90%	27	42,90%	
Idade (em anos)^2^	172	71,04 (±6,60)	63	74,29 (±8,58)	**0,008**
Escolaridade (em anos)^3^	172	2,55 (±2,73)	63	3,03 (±3,06)	0,251
Estado Civil - Casado ou com companheiro^1^	102	61,10%	36	59,00%	0,778
Renda Familiar (em reais)^3^	135	2252,00 (±1363,08)	41	1913,56 (±889,71)	0,137
Presença de Comorbidades^1^	78	45,30%	26	41,30%	0,577
Qualidade de Vida^3^	171	0,76 (±0,13)	61	0,72 (±0,15)	**0,041**
Suporte Social^3^	170	81,98 (±17,88)	59	80,66 (±19,59)	0,634
Autopercepção de Saúde ^3^	169	7,87 (±2,28)	60	6,76 (±2,34)	**0,002**

1 Teste Qui-Quadrado;

2 Teste de Mann-Whitney;

3 Teste T de Student

Ao verificar as variáveis associadas à restrição à participação, o modelo contendo apenas a avaliação subjetiva da saúde foi o único significativo [x^2^ (1) = 4,20; p < 0,04, R^2^ Nagelkerke = 0,04], indicando que a pior autopercepção de saúde é um preditor de restrição à participação relacionada à perda auditiva (OR= 0,85; IC 95%= 0,72 - 0,99).

## DISCUSSÃO

Compreende-se que a perda auditiva piora conforme o avanço do envelhecer sendo que nos indivíduos mais velhos há uma maior prevalência da restrição à participação, em decorrência da dificuldade de comunicação e diminuição da participação social^(33,34)^. Dois a cada três indivíduos que possuem qualquer nível de perda de audição irão desenvolver significativa restrição à participação em até cinco anos^(33)^. Assim, é importante conhecer os fatores relacionados a esta condição, uma vez que a perda auditiva é prevalente e progressiva na população idosa, com potencial desfecho negativo para o envelhecimento.

Estudos demonstraram que a pior qualidade de vida está relacionada às dificuldades auditivas e à restrição à participação^(8,12,33-35)^. A avaliação da qualidade de vida está associada a múltiplos aspectos pessoais (internos) e ambientais (externos): a estabilidade das relações interpessoais, bom estado de saúde e a capacidade da pessoa a se adaptar ao meio no qual está inserida^(33)^. Assim, quando a perda auditiva leva à restrição à participação, compromete o bem-estar, pode gerar estresse e solidão, impactando a qualidade de vida^(5,36,37)^. As pessoas idosas com restrição à participação estão mais propensas a terem impacto na qualidade de vida, principalmente relacionado ao fator ambiente^(8)^. Segundo a CIF, os fatores contextuais, que englobam os aspectos ambientais e pessoais, podem atuar como facilitadores ou barreiras para a funcionalidade e incapacidade de uma pessoa, destacando a importância de se considerar os fatores contextuais ao avaliar a restrição à participação e compreender o indivíduo como um todo em seu ambiente^(2)^.

Em um estudo^(34)^ foi avaliada a associação entre restrição à participação relacionada à perda auditiva e qualidade de vida em uma amostra de pessoas idosas (n= 781 homens e 950 mulheres). Para compor o teste de qualidade de vida foram considerados o bem-estar subjetivo, sintomas depressivos, solidão e funcionalidade física. Após controlar fatores de risco para presbiacusia, os autores demonstraram que a qualidade de vida se mostrou um fator significativamente associado à restrição à participação avaliada por meio do resultado do HHIE-S, mas não à pergunta única sobre a existência de problemas auditivos. Além disso, dentre o conjunto de variáveis que compuseram a avaliação da qualidade de vida, o bem-estar subjetivo foi a que mais fortemente se associou à restrição à participação em pessoas idosas, com razão de chance de 4,6 (95% CI:2,9 - 7.5), evidenciando que a baixa percepção de saúde aumenta consideravelmente as chances de restrição à participação^(34)^. Tal resultado também é evidenciado no presente estudo.

A associação entre o bem-estar subjetivo e o comprometimento auditivo também foi identificada em um estudo^(34)^. Controlada por diferentes variáveis como idade, gênero, condições de saúde e hospitalização no último ano, a restrição à participação decorrente de perda auditiva foi associada à maior probabilidade do indivíduo possuir pior autopercepção de saúde indicando uma associação independente entre a restrição à participação e os indicadores de bem-estar. Esse resultado, assim como o da presente pesquisa, demonstram que a saúde geral, bem como ela é percebida pelo indivíduo, reflete nas suas habilidades de comunicação, interação e participação social.

A avaliação subjetiva de saúde tem se mostrado um indicador importante de bem-estar em pessoas idosas, com potencial para influenciar os demais aspectos de vida das mesma^(10,38,39)^. Segundo a OMS, essa complexidade nos estados de saúde e funcionalidade das pessoas idosas suscita questões fundamentais sobre o que queremos dizer com saúde em idade mais avançada, como a medimos e como podemos promovê-la. Avaliações abrangentes desses estados de saúde são preditores de desfechos negativos melhores do que a presença de doenças individuais ou mesmo o grau de comorbidades. Realizam, portanto, um papel de indicador de saúde populacional, sendo consideradas uma das medidas vitais para avaliar os desfechos de saúde^(40)^.

Conforme o avanço do envelhecimento, o indivíduo torna-se mais suscetível às perdas físicas, psicossociais, fisiológicas e cognitivas as quais refletem nos desfechos de sua saúde e na visão negativa devido a tais mudanças em comparação a quando se era mais jovem^(41)^. Os fatores emocional e social que englobam a avaliação da restrição à participação decorrente de perda auditiva têm grande relevância à saúde física e mental de pessoas idosas e impactam na autoavaliação de saúde^(33,42)^, sensação de autonomia, controle e funcionalidade nas atividades de vida diária e, de modo geral, interferem na percepção de saúde. Assim, compreender as complexas interações entre a autoavaliação da saúde e as dimensões que influenciam a mudança na percepção de saúde pode ser a chave para o bem-estar da pessoa idosa^(43,44)^.

É importante possuir o olhar voltado ao indivíduo integralmente, levando em consideração que o mesmo está em uma diferente fase e contexto de vida e, que a sua percepção perante à própria saúde e seu bem-estar subjetivo são mecanismos relevantes para nós, enquanto profissionais da saúde, com o intuito de nos auxiliar na forma de intervir com as alterações que ocorrem no envelhecimento.

A literatura aponta que fatores socioeconômicos interferem na percepção da restrição à participação^(8)^. De maneira discordante, no presente estudo, idade, escolaridade, renda e suporte social não se diferenciaram entre os grupos com e sem restrição à participação. Ressalta-se que a amostra foi composta por pessoas idosas de uma região em vulnerabilidade social, com características socioeconômicas muito próximas, sendo uma possível explicação para a semelhança entre os grupos estudados. Entretanto, a realização deste estudo com população idosa em contexto de alta vulnerabilidade social acrescenta um diferencial na literatura à medida em que restrição à participação também é influenciada pelo meio ambiente no qual a pessoa vive, ou seja, os impactos decorrentes de uma privação auditiva também são frutos das condições impostas pela ambiência, acesso e condições de saúde^(45)^.

O presente estudo aponta a importância da autopercepção na avaliação biopsicossocial do indivíduo idoso. Outro fator relevante é da pesquisa se basear na restrição à participação relacionada à perda auditiva, tendo em vista que os exames audiométricos não avaliam o contexto ambiental e nem como os indivíduos se sentem em relação aos aspectos emocionais e sociais da comunicação. Aponta-se, por fim, que o HHIE-S é uma avaliação com maior confiabilidade, especificidade e sensibilidade para avaliar o impacto de perda auditiva na qualidade de vida comparada à pergunta única “Você tem perda auditiva?”^(35)^. Desse modo, salienta-se a importância da utilização do instrumento para a detecção dos impactos oriundos da perda auditiva.

A avaliação das capacidades intrínsecas do indivíduo e sua relação com o meio são fundamentais para conhecer a capacidade funcional e seus impactos no processo de envelhecimento saudável. Assim, a autopercepção dos aspectos de saúde e de comunicação são fatores que podem ser úteis para as ações que visem a saúde biopsicossocial e que devem ser consideradas por uma equipe multidisciplinar. O estudo aponta a importância de mais pesquisas voltadas para entender a relação entre bem-estar subjetivo e as percepções da perda auditiva como uma valiosa informação nos processos de reabilitação auditiva da pessoa idosa, favorecendo o uso, benefício e satisfação dos dispositivos de amplificação sonora.

A limitação do presente estudo refere-se à amostra de uma única cidade brasileira, em uma comunidade composta por pessoas com características sociais muito semelhantes entre si. Por outro lado, há um diferencial no que diz respeito a isso, visto que abarca uma amostra em domicílio em situação de vulnerabilidade social, em um universo de pesquisas brasileiras desenvolvidas majoritariamente no ambiente ambulatorial, ou pesquisas de campo realizadas em países ricos com outras características populacionais. Desse modo, a compreensão dos resultados provenientes de comunidades em situação de vulnerabilidade social são mais fidedignas à realidade e permitem ações que promovam a equidade.

## CONCLUSÃO

No presente estudo conclui-se que a avaliação subjetiva de saúde foi a única variável independentemente associada à restrição à participação decorrente de perda auditiva em pessoas idosas. Apesar da qualidade de vida se mostrar menor no grupo com restrição à participação, tal associação não se manteve ao controlar demais variáveis. Quanto ao suporte social , este não apresentou nenhuma relação com a restrição à participação. A relevância do estudo integra a visão da autopercepção do indivíduo sobre as suas condições de saúde e o impacto da perda auditiva, com o enfoque na restrição à participação.
